# Effects of contact tracing and nucleic acid testing on the COVID-19 outbreak in Zunyi, China: data-driven study using a branching process model

**DOI:** 10.1186/s12879-022-07967-2

**Published:** 2023-01-06

**Authors:** Jun Feng, Wenlong Zhu, Xingui Ye, Zhixi Liu, Yue Zhu, Qinyi Wu, Guanghong Yang, Weibing Wang

**Affiliations:** 1 Guizhou Provincial Center for Disease Control and Prevention, 73 Ba Ge Yan Road, Guiyang, 550000 China; 2 School of Public Health, Shanghai Institute of Infectious Disease and Biosecurity, Fudan University, 138 Yi Xue Yuan Road, Shanghai, 200032 China; 3 Key Laboratory of Public Health Safety of Ministry of Education, Fudan University, 138 Yi Xue Yuan Road, Shanghai, 200032 China

**Keywords:** COVID-19, Outbreak, Vaccine, Non-pharmaceutical intervention (NPI), Branching process model

## Abstract

**Background:**

During October 2021, China experienced localized outbreaks of COVID-19 in many cities. We analyzed the small local outbreak in Zunyi (Guizhou Province), a major city in southwestern China, and modeled the effects of different interventions on this outbreak.

**Methods:**

Data on infections and contacts, provided by the Health Commission of Guizhou Province, were used to analyze the epidemiological characteristics of the outbreak and calculate the effectiveness of vaccination. A branching process model was used to simulate the outbreak. This model considered the time interval from exposure of the initial case to confirmation, the number of potential infections caused by the initial case, and the effects of the different interventions.

**Results:**

From 18 to 25 October 2021, there were 12 patients with COVID-19 in Zunyi. Overall, the average age was 67.17 years-old, 8 patients were females, and 1 patient had an asymptomatic infection. The effectiveness of two-dose inactivated vaccine against SARS-CoV-2 infection was 16.7% (95% CI: 2.8% to 99.7%). The initial case was infected on 11 or 12 October 2021, 6.40 (95% CI: 6.37, 6.42; IQR: 4.92, 7.63) days before confirmation while the travelling in Lanzhou (Gansu Province). There were 10.07 (95% CI: 10.04, 10.09; IQR: 7.86, 11.93) potential secondary cases. When the effective vaccine coverage reached 60%, the probability of cumulative cases exceeding 20 was less than 8.77%, even if contact tracing was relaxed or eliminated. However, if the probability of tracing contacts decreased, earlier initiation of nucleic acid testing was necessary to control the outbreak.

**Conclusions:**

The COVID-19 outbreak in Zunyi was controlled quickly due to moderately effective vaccine coverage and rapid contact tracing. For controlling localized outbreaks, vaccination and contact tracing seemed to be more effective than massive nucleic acid testing in the initial phase of transmission. However, if there is low effective vaccine coverage or insufficient contact tracing, nucleic acid testing should start earlier.

**Supplementary Information:**

The online version contains supplementary material available at 10.1186/s12879-022-07967-2.

## Background

Coronavirus disease 2019 (COVID-19), which is caused by the SARS-CoV-2 virus, emerged in Wuhan during December 2019, and the WHO subsequently characterized this disease as a pandemic [1]. As of 10 December 2021, there were 267 million confirmed cases and 5.28 million deaths worldwide [2]. Although China successfully controlled the first-wave of the COVID-19 epidemic by the end of March 2020, it faced the challenge of resurgence due to foreign visitors [3, 4]. From 2020, there were several sporadic and localized outbreaks in the regions of Beijing [5], Urumqi [6], Dalian [7], Shanghai [8], Shijiazhuang [9], Ruili [10], Nanjing, Yangzhou [11], and Inner Mongolia. From the first case in late 2019 until 10 December 2021, there were 99,604 patients known to have COVID-19 in mainland China [12], and half of them were in several localized outbreaks.

Concerted global efforts led to the development of several COVID-19 vaccines. China first administered a COVID-19 vaccine (Beijing CNBG BBIBP-CorV) on 30 December 2020 [13], and subsequently provided vaccines free-of-charge to all citizens [14]. As of 11 December 2021, 2.59 billion doses of COVID-19 vaccines were administered in mainland China [15]. However, China’s large and heterogeneous population has made it difficult to achieve herd immunity. In addition, SARS-CoV-2 has continued to evolve, and many variants have emerged throughout the world. As of 11 December 2021, the WHO designated five SARS-CoV-2 variants of concern (VOCs; Alpha, Beta, Gamma, Delta, and Omicron) [16]. As the virus evolved, its transmissibility seemed to increase and vaccine effectiveness seemed to decrease [16, 17]. Some studies suggested that before achieving high vaccine coverage, non-pharmaceutical interventions (NPIs) were needed to prevent outbreaks and resurgences [18–20].

On 16 October 2021, two tourists tested positive for COVID-19 in Jiayuguan City (Gansu Province) after previously visiting multiple cities in Inner Mongolia and Gansu Province. Within 12 days, the epidemic spread rapidly to 25 cities in 12 provinces and municipalities. More than 300 new positive cases were reported nationwide, and more than 280 cases were directly or indirectly linked to the epidemics in Inner Mongolia and Gansu Province.

Zunyi (Guizhou Province) is a major city in southwestern China that has a population of 6.6 million and 15 administrative districts. On 18 October 2021, Zunyi experienced a local outbreak of COVID-19 that was related to the epidemic in Gansu Province. We used data provided by the Health Commission of Guizhou Province to analyze the transmission chain of this outbreak and to estimate the effectiveness of COVID-19 vaccination. In particular, we constructed a branching process model to analyze major characteristics of this outbreak, such as the different time intervals from exposure to diagnosis and different number of infections caused by the initial case. We also examined the combined effects of vaccination and NPIs in controlling this localized COVID-19 outbreak.

## Methods

### Data collection

Data on people who had COVID-19 were provided by the Health Commission of Guizhou Province, which were also public publicly available at http://wjw.guizhou.gov.cn/. These data included demographic characteristics (age and sex), dates of confirmed diagnosis and isolation, information on vaccination, records of movement, and identity of close contacts who had COVID-19. The demographic characteristics and vaccination status of contacts in the locus of transmission—a mahjong room—were also included. All cases were diagnosed according to the Diagnosis and Treatment Protocol for Coronavirus Pneumonia (Trial Version 8) from the National Health Commission of China. Close contacts in the mahjong room are individuals who played mahjong with the initial case (P1) in this room on 18 October 2021. A matched case–control study was designed to estimate the effectiveness of inactivated vaccine. For each case, we matched four close contacts with the same sex and age plus or minus 5 years. Control group was selected among close contacts of cases who had a high probability of contracting the virus. Waiver of informed consent for collection of epidemiological data from patients with COVID-19 was granted by the Health Commission of Guizhou Province as part of the infectious disease outbreak investigation.

### Main control measures during the local outbreak

On 18 October 2021, one person (P1) with a positive nucleic acid test was identified during the routine nucleic acid testing of people from high-risk areas who returned to Zunyi [21]. This patient received treatment in a designated hospital and all close contacts were quickly traced and quarantined. All locations where these contacts had travelled were thoroughly disinfected. On 23 October 2021, the government of Zunyi decided to organize three-rounds of nucleic acid testing from 23 to 27 October in the central areas of Zunyi, including Honghuagang District, Huichuan District, Bozhou District, and Xinpuxin District [22].

### Model structure

Based on Hellewell et al. [23], a branching process model that considered vaccination and nucleic acid testing was used to analyze COVID-19 transmission and control. The model was initiated using one symptomatic case (P1) to represent the onset of a new outbreak. This initial symptomatic case was isolated after symptom onset after a delay in time. The isolation was assumed to be 100% effective in preventing further transmission. The number of potential secondary infections was derived from a negative binomial distribution with a mean equal to the reproductive number (R_0_). Each potential secondary infection was assigned an exposure time drawn from the serial interval, and new secondary cases were created if they were unvaccinated and the index case was not isolated at the time of infection.

Each newly infected case was identified using close contact tracing or nucleic acid testing. Newly infected cases were identified by contact tracing with a probability *p* and were isolated immediately. Cases missed by contact tracing could potentially be detected using extensive nucleic acid testing. During a round of nucleic acid testing, cases missed due to a false negative result were detected and confirmed during a subsequent round, and were then isolated immediately. For each case, there was an assumed 1 to 3 days window during which cases always had negative nucleic acid test results. In addition, each case had an independent probability of having an asymptomatic infection, and therefore could only be detected by nucleic acid testing (not contact tracing).

**Fig. 1:**
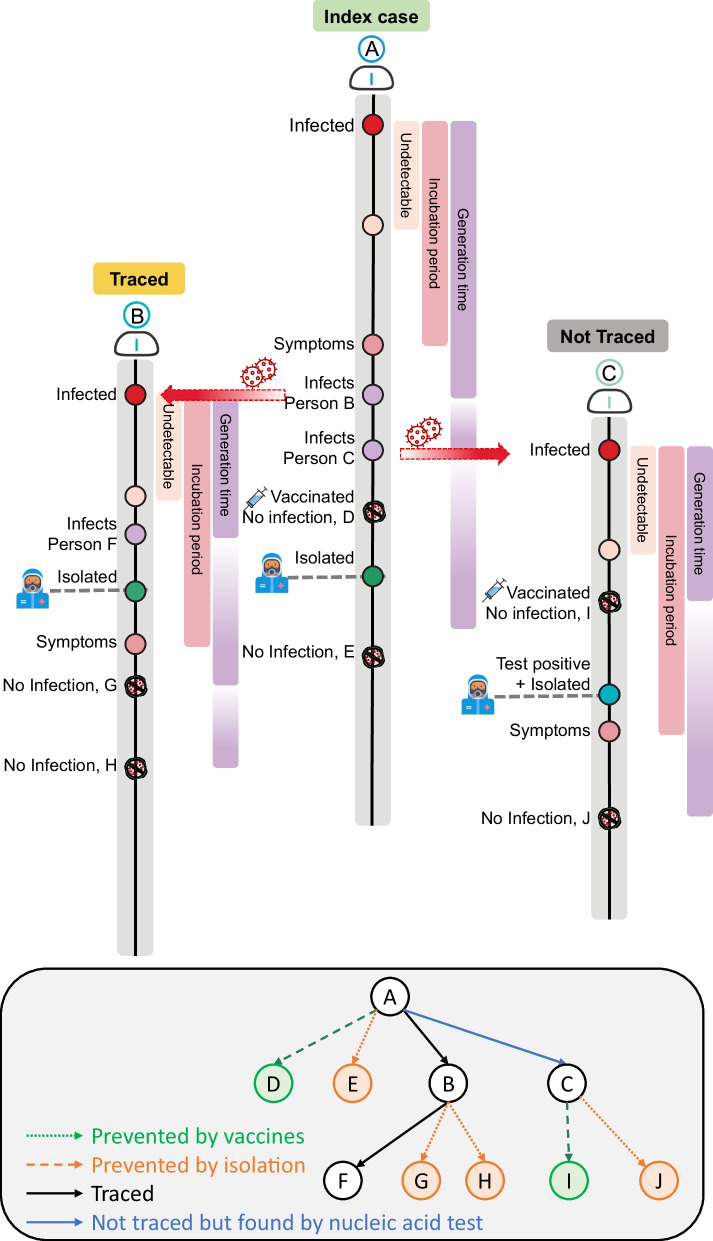
Design and a hypothetical example of the branching process model

A simple hypothetical example illustrates these interventions in the model (Fig. 1). In particular, case A had contact with and potentially infected four other individuals (cases B, C, D and E). Case D was protected by vaccination, and case E was protected by isolating cases A, so case A was responsible for two new infections (cases B and C). Even though case B was identified by contact tracing, he/she still infected case F, but not cases G and H because they were protected by isolation. Case C was not identified by contact tracing, but was identified by nucleic acid testing and then isolated. Thus, cases I and J were not produced.

### Model parameters and scenarios

To simulate the transmission and control of the outbreak in Zunyi, the model was initiated with one case. To model transmission in the mahjong room, there were assumed to be 8 secondary infections (7 who played mahjong with the initial case and 1 who was a family member of the initial case). In a highly localized outbreak, almost all cases are detected by contact tracing, so the probability of being traced was assumed to be 90%. The vaccine coverage in Zunyi was 76.22% [24], and vaccine effectiveness was 59.0% [17]. Therefore, the effective vaccine coverage in the model was 44.97%. Three rounds of massive nucleic acid testing were implemented in central areas of Zunyi on 23, 25, and 27 October 2021 [22] (corresponding to 5, 7, and 9 days after the first case was confirmed). The sensitivity of the nucleic acid testing ranged from 72.44 to 97.27% [25], and was assumed to be 70% in the model. The incubation period was 4.4 days (log-normal distribution, mean value = 4.4 days, standard deviation = 1.9 days) [26], R_0_ was 5 [27], and the percentage of asymptomatic infections was 8.56%. These parameters were set to simulate the baseline scenario, as a model of the actual transmission of COVID-19 in Zunyi. In sensitivity analysis, these parameters were altered for modeling different scenarios (Additional file 1: Table S1 [17, 22, 24, 26–28]). All analyses were simulated 20,000 times. The construction of the model and simulation were conducted using R project version 4.1.1 [29].

### Statistical analysis

All infected patients were included in the statistical analysis. The epidemiological analysis considered the dates of positive nucleic acid tests, the transmission chain, and the distribution of the contacts in the mahjong room. The Wilcoxon test was used to analyze the difference in mean age of case and control groups, and the χ^2^ test or Fisher’s exact test was used to analyze other differences of these two groups. The effectiveness of vaccine (VE) was estimated to be VE = (1-OR) × 100%, where the odds ratio (OR) was derived from a conditional logistic regression model. The level of statistical significance was defined as P < 0.05.In the branching process model, the time/date of cases were infected and confirmed could be obtained from each simulation. Thus, the interval between infected and confirmed could be calculated. The number of potential infections was also calculated from the model. Based on these data, the Gamma distribution of the interval from exposure to confirmation of P1 and the number of potential infections after P1 was confirmed were estimated using a maximum likelihood method. These data were obtained from a branching process model with 20,000 simulations.

The statistical analysis, estimation of the interval from exposure to confirmation, and the number of potential infections were determined using the MASS [30] packages in R project version 4.1.1.

## Results

**Fig. 2:**
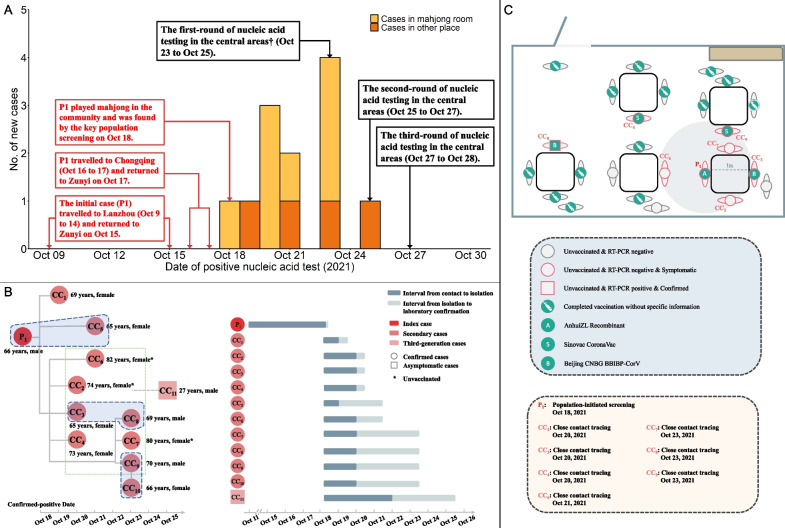
Number of new cases on different dates (**A**). Potential transmission chains (**B**). Distribution of clustered cases in the mahjong room (**C**). †Central areas included Honghuagang District, Huichuan District, Bozhou District, Xinpuxin District. Transmission chains (**B**) and distribution of clustered cases in the mahjong room (**C**) was obtained from the epidemiological data from Health Commission of Guizhou Province

From 18 to 25 October 2021, there were 12 patients with COVID-19 in Zunyi (Table 1, Fig. 2A). The average age was 67.2 years-old (median: 69.0, Inter-Quartile Range (IQR): 65.8, 73.2), 8 patients were females, 1 patient had an asymptomatic infection, 3 patients were unvaccinated (Table 1). Based on the matched case control study, we estimated the effectiveness of two-dose inactivated vaccine against SARS-CoV-2 infection was 16.7% (95% CI: 2.8% to 99.7%). We used epidemiological data to infer the potential transmission chain among these patients (Fig. 2B). Thus, the initial case (P1) was in Lanzhou (Gansu Province) on 9 to 14 October, was in the Chongqing municipality on 16 to 17 October, and returned to Zunyi on 17 October. P1 played mahjong in the local community and was identified by the routine nucleic acid testing performed on people from high-risk areas on 18 October. Eight close contacts (CC2–CC4 and CC6–CC10) were infected by P1 when they played mahjong. CC5 was a family member who travelled with P1, so could have been infected by another person or by P1; CC1 also travelled with P1 to Chongqing, so could also have been infected by another person or by P1. CC10 and CC11 were infected by other cases in the mahjong room (CC2–CC4 or CC6–CC10). Figure 2C presented the distribution of individuals in the mahjong room. This room had 25 individuals, the average age was 73.9 years-old (median: 73.0, IQR: 72.0, 80.0), and there were 15 females (60.0%). Eight individuals (P1, CC2–CC4 and CC6–CC10) were infected and 20 individuals (80.0%) were vaccinated.

**Table 1:** Characteristics of patients and their matched controls

Characteristics	Overall	Control group	Case group	P-value
N	60 (100%)	48 (80%)	12 (20%)	
Age, years	66.0 (60.0, 70.0)	65.0 (58.0, 70.0)	69.0 (65.8, 73.2)	0.095
*Sex*				
Female	40 (66.7%)	32 (66.7%)	8 (66.7%)	> 0.999
Male	20 (33.3%)	16 (33.3%)	4 (33.3%)	
*Vaccine status*				
Unvaccinated	5 (8.3%)	2 (4.2%)	3 (25.0%)	0.049
Vaccinated	55 (91.7%)	46 (95.8%)	9 (75.0%)	
*Dose*				
0	5 (8.3%)	2 (4.2%)	3 (25.0%)	0.172
1	1 (1.7%)	1 (2.1%)	0 (0.0%)	
2	52 (86.7%)	43 (89.6%)	9 (75.0%)	
3	2 (3.3%)	2 (4.2%)	0 (0.0%)	

Values are given as N (%) or median (Inter-Quartile Range, IQR). ^†^*P* value was calculated by the χ^2^ test or the Wilcoxon test

**Fig. 3:**
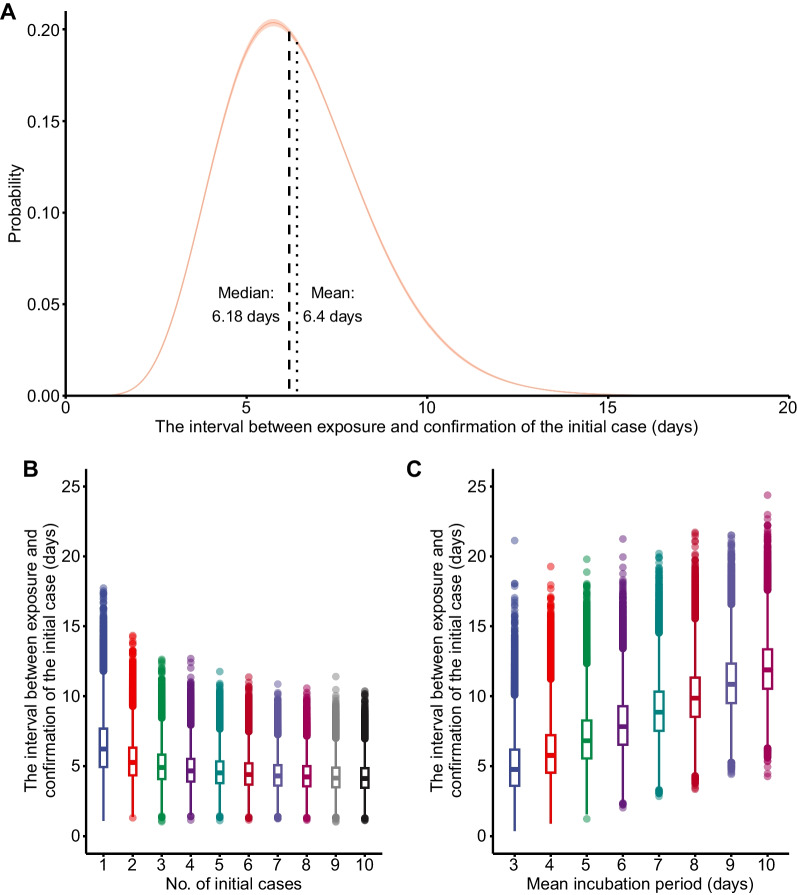
Gamma distribution of the interval between exposure and confirmation of the initial case (**A**). Effect of the number of initial cases on the interval (**B**). Effect of mean incubation period on the interval (**C**)

Assuming the mean incubation period was 4.4 days (baseline scenario for COVID-19 in Zunyi), after 20,000 simulations the branching process model estimated that the initial case (P1) was infected 6.40 days (95% CI: 6.37, 6.42; IQR: 4.92, 7.63) before being detected. In other words, P1 was likely infected on 11 or 12 October when he was in Lanzhou. The time interval from being infected to detected had a Gamma distribution (α [shape] = 9.749, 95% CI: 9.589, 9.909; β [rate] = 1.524, 95% CI: 1.496, 1.552; Fig. 3A). Sensitivity analysis indicated that this interval decreased from 6.430 days (median: 6.218, IQR: 4.395, 7.677) to 4.194 days (median: 4.108, IQR: 3.444, 4.849) as the number of initial infections increased from 1 to 10 (Fig. 3B). This interval increased from 5.020 days (median: 4.758, IQR: 3.586, 6.181) to 12.009 days (median: 11.881, IQR: 10.525, 13.350) when the mean incubation increased from 3 to 10 days (Fig. 3C). The R_0_, the effective vaccine coverage, the probability of contacts traced, and other interventions had no effect on this interval (Additional file 1: Figure S1).

**Fig. 4:**
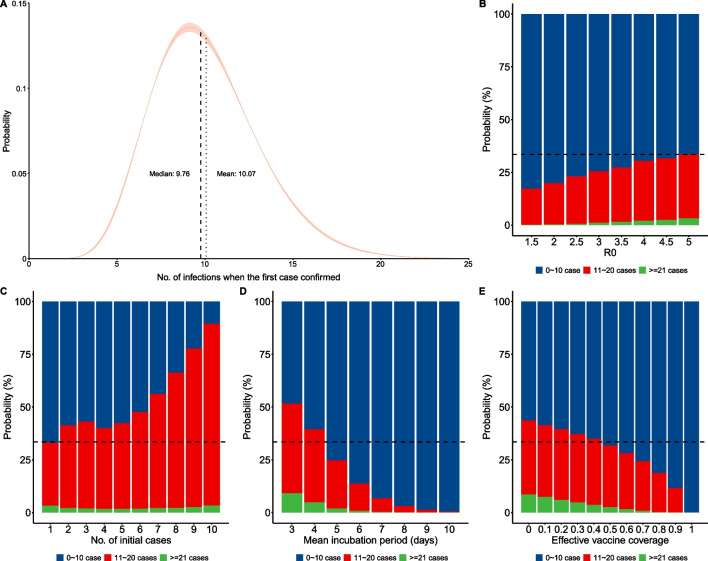
Gamma distribution of the number of potential infections when there was a single case (**A**). Effect of R_0_ (**B**), number of initial cases (**C**), mean incubation period (**D**), and effective vaccine coverage (**E**) on the number of infections. Horizontal black dotted lines (**B–E**) refer to the baseline scenario, in which 66.48% of the simulations led to fewer than 10 potential infections

We estimated there were 10.07 (95% CI: 10.04, 10.09; IQR: 7.86, 11.93) potential infections before identification of P1 (Fig. 4A). Among the 20,000 simulations, 66.48% led to fewer than 10 potential infections, and 3.16% led to more than 20 potential infections. The number of potential infections increased as R_0_ increased and as the number of initial cases increased (Fig. 4B, C), but decreased as the incubation period increased and as the effective vaccine coverage increased (Fig. 4D, E). The percentage of simulations with fewer than 10 infections declined from 82.79 to 66.48% as R_0_ increased from 1.5 to 5, and declined from 66.96 to 10.85% as the number of initial cases increased from 1 to 10 (Fig. 4B, C). When the incubation period increased from 3 to 10 days, the percentage of simulations that led to fewer than 10 infections increased from 48.44 to 99.50%; this percentage increased from 56.45% to 100% when the effective vaccine coverage increased from 0 to 100% (Fig. 4D, E). The percentage of asymptomatic infections, the probability of contacts being traced, and the start time of nucleic acid testing had small effects on the number of potential infections (Additional file 1: Figure S2).

**Fig. 5:**
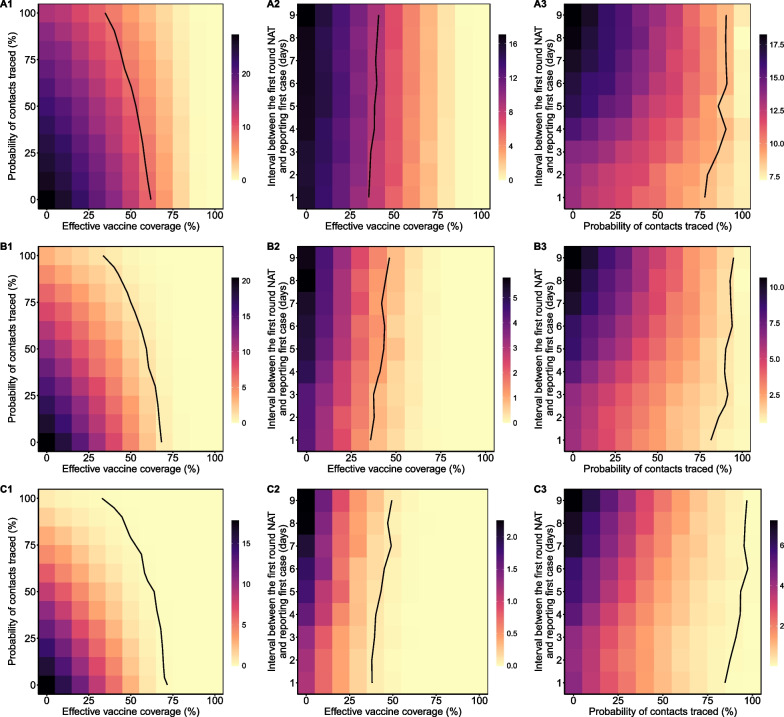
Probability (%) of cumulative cases exceeding 20 (**A1**–**A3**), 50 (**B1**–**B3**), and 100 (**C1**–**C3**) under different effective vaccine coverage (**A1**–**C1**), probability of tracing contacts (**A2**–**C2**), and onset of nucleic acid testing (**A3**–**C3**). The solid black lines are isopleths corresponding to 8.77% (**A1**–**A3**), 1.05% (**B1**–**B3**) and 0.10% (**C1**–**C3**)

In the baseline scenario, we also calculated the probability of cumulative cases more than 20 (8.77%), more than 50 (1.05%), and more than 100 (0.10%). These findings indicated that the outbreak would not be serious. During the outbreak, the probability of the cumulative number of cases exceeding 20/50/100 increased as R_0_ increased and as the number of initial cases increased, but decreased as the incubation period decreased, as effective vaccine coverage increased, and as the probability of tracing contacts increased. The proportion of asymptomatic infections and the start time of the nucleic acid testing had small effects on these probabilities (Fig. 5, Additional file 1: Figure S3).

Thus, to control this localized outbreak, vaccination and close contact tracing appeared to be more effective than massive nucleic acid testing. If massive nucleic acid testing started 5 days after the first case was confirmed and the effective vaccine coverage was 60%, the probability of cumulative cases exceeding 20 was less than 8.77%, even if close contact tracing was relaxed or cancelled (Fig. 5 A1). In this scenario, when the effective vaccine coverage reached 70%, the probability of cumulative cases exceeding 50 was 1.05%, and the probability of cumulative cases exceeding 100 was 0.10% (Fig. 5 B1, C1). When the effective vaccine coverage reached 50% or higher, and high-intensity close contact tracing was maintained but nucleic acid testing started later, the probability of cumulative cases exceeding 20 was less than 8.77%, the probability of exceeding 50 was less than 1.05%, and the probability of exceeding 100 was less than 0.10% (Fig. 5 A2, B2, C2). However, if the probability of tracing contacts decreased, it was necessary to start nucleic acid testing earlier to achieve better control of the outbreak (Fig. 5 A3, B3, C3).

## Discussion

Our analysis of the travel records of the initial case (P1) of COVID-19 and estimations from the model indicated that this individual was infected during his time in Lanzhou. P1 subsequently spread COVID-19 to other susceptible individuals while playing mahjong in Zunyi, precipitating to a localized cluster of community transmission. The SARS-CoV-2 virus has evolved during the pandemic, and variants with high transmission rate tend to have shorter incubation periods [16, 31]. Based on our results, these changes in the characteristics of the virus would increase the number of infections during an outbreak and would also increase the probability of a large outbreak. Thus, more strict interventions are likely needed to avoid resurgences and to prevent outbreaks.

We estimated the effectiveness of two-dose inactivated vaccine against SARS-CoV-2 infection was 16.7% (95% CI: 2.8% to 99.7%), lower than the results of Li et al. [17]. The small sample size is the main limitation for the estimation of vaccine effectiveness. Studies that examined more individuals from different cities are needed to provide a more reliable estimate of vaccine effectiveness. An effective vaccine is considered key for preventing further morbidity and mortality from COVID-19 [32]. Previous studies suggested that widespread vaccination can allow relaxation of the need for stringent NPIs [18–20, 33]. Our study of a highly localized outbreak indicated that if the effective vaccine coverage reached 60%, the probability of cumulative cases exceeding 20 was less than 10%, even if close contact tracing was relaxed or eliminated. These results indicated that high effective vaccine coverage helped to minimize the scale of the outbreak and made up for deficiencies of NPIs, such as the false-negative test results, the high cost of nucleic acid testing, and missing contacts. Thus, increasing vaccine coverage plays a significant role in preventing resurgence and in minimizing the scale of an outbreak.

Massive nucleic acid testing can play a critically important role in sustaining the containment of SARS-CoV-2 [34, 35]. Adherence of community residents, cross-infection control, and testing capacity all influence the effectiveness of massive nucleic acid testing campaigns. However, compared with more intensive contact tracing, massive nucleic acid testing did not seem to provide sufficient protection in our model. Our results indicated that if the probability of contact tracing was less than 50%, there would be more cases, even if massive nucleic acid testing started very early. Based on the “Zero-COVID” policy, rapid and effective contact tracing can reduce the number of cases during the early stage of an outbreak, and can make the outbreak easier to control overall [23, 36]. According to our simulations of a localized outbreak, if the probability of contact tracing is high, the time when nucleic acid testing begins had limited impact on the scale of the outbreak. However, effective contact tracing depends on high reporting accuracy, adherence, coverage, and speed [36], and these can be difficult to achieve during large outbreaks. If the effectiveness of contact tracing decreases, nucleic acid testing should start early or other NPIs should be used to minimize the scale of the outbreak, especially large-scale community transmission.

The study has some limitations. First, there may be biases in the estimation of vaccine effectiveness due to our small sample size. The effectiveness of vaccines against different SARS-CoV-2 variants must be considered when attempting to control subsequent outbreaks. Thus, more attention should be given to the effectiveness of vaccines and the impacts of different variants on their effectiveness. Second, due to lack of the relevant parameters, we were unable to consider the effects of age in the current model. Combined with the age-specific contact data in a subsequent study might be able to consider this parameter and thereby improve the versatility of the model [37]. Third, this study was based on a highly localized outbreak. Application of our model to large-scale outbreaks and long-lasting epidemics should be considered with caution.

## Conclusions

In conclusion, based on the “Zero-COVID” policy, when maintaining high effective vaccine coverage, rapid and effective contact tracing appears sufficient to control a highly localized outbreak in city such as Zunyi, which has a population of 6.6 million. For other scenarios, such as large-scale community outbreaks or low effective vaccine coverage, early adoption of massive nucleic acid testing and other interventions should be considered.

## Supplementary Information

**Additional file 1: Table S1.** Initial baseline values and values used in the sensitivity analysis of the branching process model. **Figure S1.** Sensitivity analysis of the interval of initial case from exposure to confirmation. **A**, **B**, **C**, **D** and **E** was the impact of R_0_, proportion of asymptomatic infections, effective vaccine coverage, probability of contact traced and start time of nucleic acid test on the interval respectively. **Figure S2.** Sensitivity analysis of the number of potential infections for different proportions of asymptomatic infections (**A**), different probability of contact tracing (**B**), and different start time of nucleic acid testing (**C**). The horizontal black dotted lines indicate the baseline scenario (66.48% simulations with potential infections less than 10) using parameters in Table S1. **Figure S3.** Probability of cumulative cases exceeding 20 (blue), 50 (red), and 100 (green) for different values of R_0_ (**A**), different numbers of initial cases (**B**), different mean incubation period (**C**), different proportion of asymptomatic infections (**D**), different effective vaccine coverage (**E**), different probability of contact tracing (**F**), and different start time of nucleic acid testing (**G**). The vertical grey dotted lines indicate the baseline scenario, using parameters in Table S1.

## Data Availability

The datasets used and/or analyzed during the current study are available from the corresponding author on reasonable request.

## Abbreviations

COVID-19: Coronavirus disease 2019
SARS-CoV-2: Severe acute respiratory syndrome coronavirus 2
WHO: World Health Organization
VOCs: Variants of concern
NPIs: Non-pharmaceutical interventions
IQR: Inter-quartile range
NAT: Nucleic acid test
95% CI: 95% Confidence interval
