# Supplementary material for: Effects of contact tracing and nucleic acid testing on the COVID-19 outbreak in Zunyi, China: data-driven study using a branching process model
Source: BMC Infect Dis. 2023 Jan 6;23:10. doi: 10.1186/s12879-022-07967-2 (PMC9821355; doi:10.1186/s12879-022-07967-2)
Supplement: Supplementary file 1 — Additional file 1: Table S1. Initial baseline values and values used in the sensitivity analysis of the branching process model. Figure S1. Sensitivity analysis of the interval of initial case from exposure to confirmation. A, B, C, D and E was the impact of R0, proportion of asymptomatic infections, effective vaccine coverage, probability of contact traced and start time of nucleic acid test on the interval respectively. Figure S2. Sensitivity analysis of the number of potential infections for different proportions of asymptomatic infections (A), different probability of contact tracing (B), and different start time of nucleic acid testing (C). The horizontal black dotted lines indicate the baseline scenario (66.48% simulations with potential infections less than 10) using parameters in Table S1. Figure S3. Probability of cumulative cases exceeding 20 (blue), 50 (red), and 100 (green) for different values of R0 (A), different numbers of initial cases (B), different mean incubation period (C), different proportion of asymptomatic infections (D), different effective vaccine coverage (E), different probability of contact tracing (F), and different start time of nucleic acid testing (G). The vertical grey dotted lines indicate the baseline scenario, using parameters in Table S1. [file 12879_2022_7967_MOESM1_ESM.docx]

**Additional file for**

**Effects of contact tracing and nucleic acid testing on the COVID-19 outbreak in Zunyi, China: data-driven study using a branching process model**

Jun Feng^1†^, Wenlong Zhu^2†^, Xingui Ye^1^, Zhixi Liu^2^, Yue Zhu^2^, Qinyi Wu^1^, Guanghong Yang^1*^, Weibing Wang^2, 3*^

1 Guizhou Provincial Center for Disease Control and Prevention, 73 Ba Ge Yan Road, Guiyang 550000, China.

2 School of Public Health, Shanghai Institute of Infectious Disease and Biosecurity, Fudan University, 138 Yi Xue Yuan Road, Shanghai 200032, China.

3 Key Laboratory of Public Health Safety of Ministry of Education, Fudan University, 138 Yi Xue Yuan Road, Shanghai 200032, China.

† These authors contributed equally to this work.

* Corresponding authors:

Dr. Guanghong Yang

Guizhou Provincial Center for Disease Control and Prevention, 73 Ba Ge Yan Road, Guiyang 550004, China (e-mail: ghyang_gzmu@outlook.com).

Dr. Weibing Wang

School of Public Health, Shanghai Institute of Infectious Disease and Biosecurity, Key Laboratory of Public Health Safety of Ministry of Education, Fudan University, 138 Yi Xue Yuan Road, Shanghai 200032, China (e-mail: wwb@fudan.edu.cn).

**This file includes:**

Table S1

Figure S1 to Figure S3

Table S1. Initial baseline values and values used in the sensitivity analysis of the branching process model.

| **Parameters** | **Initial value/**  **distribution** | **Sensitivity analyses** | **Source of initial value** |
| --- | --- | --- | --- |
| Incubation period | Log-normal distribution  Mean=4.4, sd=1.9 | Mean: 3 to 10  difference=1 | Ref [26] |
| Time from symptom onset to isolation | Weibull distribution  Shape=1, scale=1 | - | Assumed |
| Undetectable window | Normal distribution>0  Mean=2, sd=1 | - | Assumed, Ref [28] |
| Number of initial cases | 1 | 1 to 10  difference=1 | Assumed |
| Reproduction number (R_0_) | 5 | 1.5 to 5  difference = 0.5 | Ref [27] |
| R_0_ after isolation | 0 | - | Assumed |
| Cases isolated once identified | 100% | - | Assumed |
| Isolation effectiveness | 100% | - | Assumed |
| Probability of contacts traced (*p*) | 90% | 0% to 100%  difference=10% | Calculated |
| Proportion of asymptomatic infections | 8.56% | 0% to 50%  difference=10% | Calculated |
| Effective vaccine coverage | 44.97% | 0% to 100%  difference=10% | Ref [17, 24] |
| Start time of the three rounds of NAT after confirmation of the first case | 5, 7, 9 | (5, 7, 9) ± 4 | Ref [22] |

NAT: nucleic acid testing; sd: standard deviation.


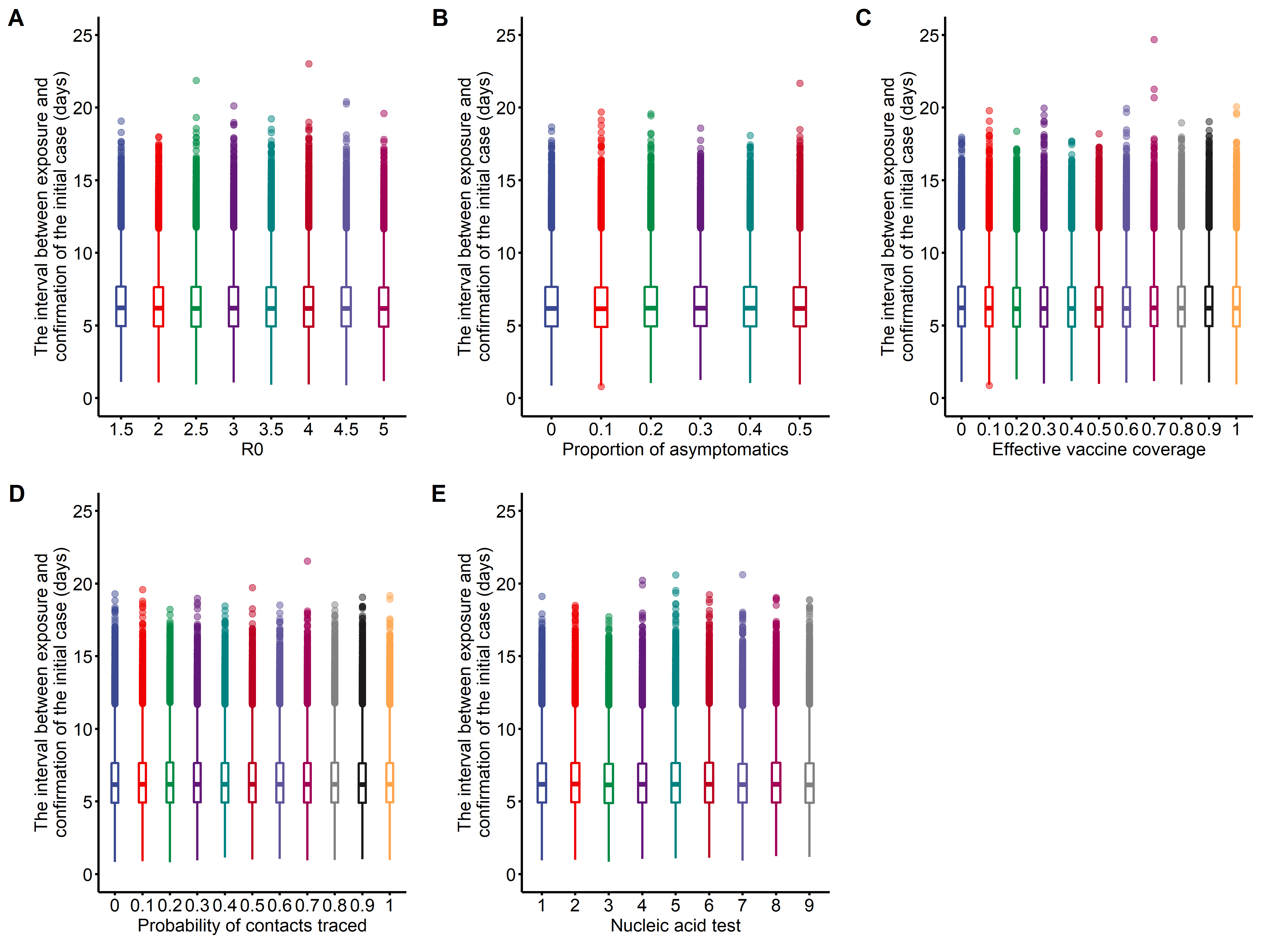
Figure S1. Sensitivity analysis of the interval of initial case from exposure to confirmation. A, B, C, D and E was the impact of R_0_, proportion of asymptomatic infections, effective vaccine coverage, probability of contact traced and start time of nucleic acid test on the interval respectively.


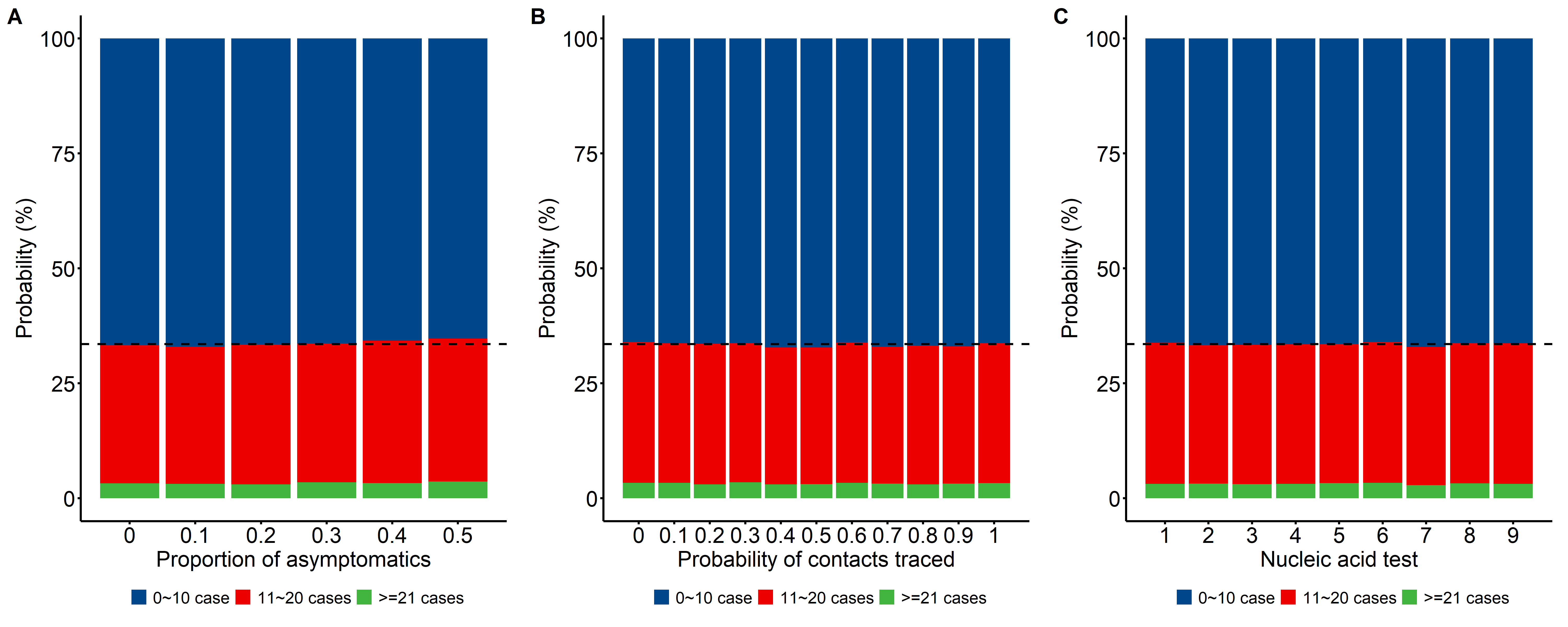
Figure S2. Sensitivity analysis of the number of potential infections for different proportions of asymptomatic infections (A), different probability of contact tracing (B), and different start time of nucleic acid testing (C). The horizontal black dotted lines indicate the baseline scenario (66.48% simulations with potential infections less than 10) using parameters in Additional Table S1.


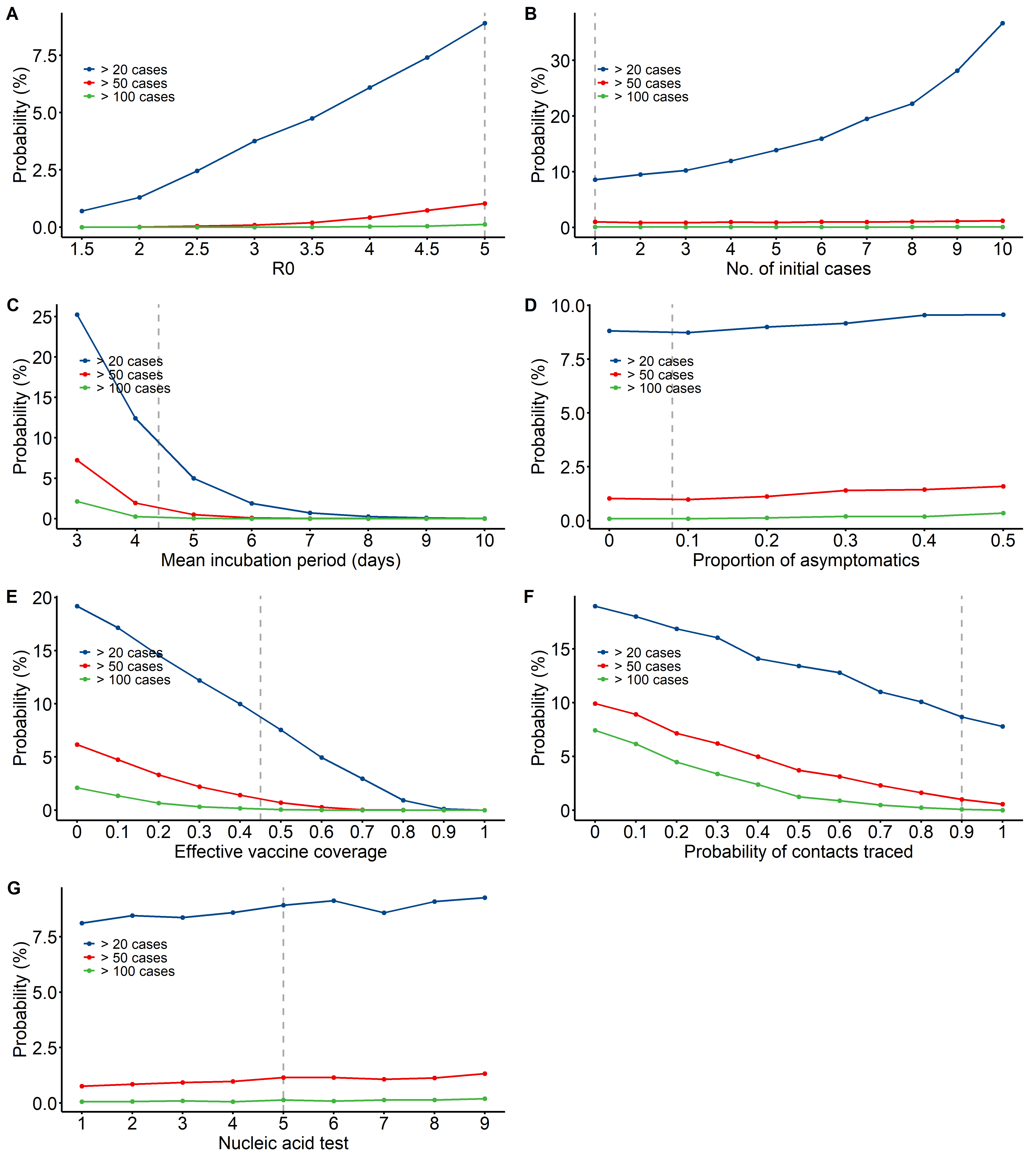
Figure S3. Probability of cumulative cases exceeding 20 (blue), 50 (red), and 100 (green) for different values of R_0_ (A), different numbers of initial cases (B), different mean incubation period (C), different proportion of asymptomatic infections (D), different effective vaccine coverage (E), different probability of contact tracing (F), and different start time of nucleic acid testing (G). The vertical grey dotted lines indicate the baseline scenario, using parameters in Additional Table S1.
